# Effects of feeding black soldier fly larvae with yellow wine lees on growth performance, nutrient composition, and gut microbiota

**DOI:** 10.3389/fvets.2026.1764177

**Published:** 2026-07-29

**Authors:** Jia Zhao, Xiang Li, Zeyuan Lian, Ruiqi Ning, Junlin Ru, Xiaoxia Liu, Jianwei Hao

**Affiliations:** 1 Department of Biological Science and Technology, Jinzhong University, Jinzhong, China; 2 Engineering Technology Research Center of Solid-State Brewing in Colleges and Universities of Shanxi Province, Jinzhong, China; 3 School of Food Science and Engineering, Shanxi Agricultural University, Jinzhong, China

**Keywords:** black soldier fly larvae, growth performance, gut bacteria, nutritional composition, yellow wine lees

## Abstract

**Introduction:**

Yellow wine lees (YWL) are a nutrient-rich by-product of yellow wine brewing and have potential as a feed substrate for black soldier fly larvae (BSFL, *Hermetia illucens*); however, their effects on larval growth, nutrient composition, and gut microbiota remain unclear.

**Methods:**

Six diets containing 0% (T0), 10% (T1), 20% (T2), 30% (T3), 40% (T4), and 50% (T5) YWL (w/w) were fed to four-day-old larvae for 16 days until the prepupal stage. Larval average dry weight, feed reduction rate, crude protein (CP), and ether extract (EE) were measured; gut bacterial communities were characterized by 16S rRNA high-throughput sequencing.

**Results:**

Larvae fed the T3 diet (30% YWL) achieved the highest average dry weight (83.69 mg) and feed reduction rate (68.49%); inclusion levels of 40% or higher significantly inhibited larval growth (*p* < 0.05). CP content peaked in the moderate-inclusion groups (T2 and T3; 47.96–48.37%), while EE increased with the YWL inclusion level. Inclusion of 10–30% YWL maintained microbial species richness, whereas inclusion of 40–50% caused species loss and community imbalance. Moderate inclusion increased the relative abundance of *Firmicutes*, notably *Terrisporobacter*, which correlated positively with growth indicators; conversely, high inclusion enriched *Fusobacterium* and *Lachnoclostridium*, correlating with growth inhibition and fat accumulation (*p* < 0.05).

**Discussion:**

Replacing cornmeal and wheat bran with 20–30% YWL improved gut microbial structure, BSFL growth efficiency, and larval crude protein content. In contrast, inclusion levels of 40% or higher, accompanied by dietary acidification and ethanol accumulation, induced gut microbial dysbiosis and suppressed larval growth.

## 1 Introduction

This study addresses the urgent need to valorize organic wastes in the context of global population growth and tightening resource constraints (1). The black soldier fly (*Hermetia illucens* L.) is an established model saprophagous insect: it exhibits rapid development, broad feeding habits, and high conversion efficiency, and has therefore become a leading species for the bioconversion of organic residues (2, 3). Its larvae can transform low-value biomass-such as food waste, livestock manure and agricultural by-products-into protein-and lipid-rich biomass, making them promising as a high-protein feed ingredient and offering a sustainable route to mitigate both waste pollution and protein scarcity (4, 5). Consequently, large-scale rearing of *H. illucens* larvae represents a promising green-industry avenue, and optimizing diet composition to improve larval growth and nutritional value is key to scaling up industrial production (6). Yellow wine lees, the principal by-product of Chinese yellow wine production, are generated in large quantities annually; improper disposal not only wastes resources but may also pose environmental risks (7). Various distillery residues (for example, baijiu lees and brewer’s spent grain) have been evaluated as animal feeds, with variable effects depending on residue type and host species. For instance, when black soldier fly larvae were fed diets including okara, maize distiller’s grain, brewer’s grains and a control hen diet, larvae receiving the hen diet and maize distiller’s grain attained the highest final weights (2.29 and 1.97 g, respectively) (8). In contrast, Ferri et al. (9) reported that brewer’s spent grain increased larval weight in mealworms (*Tenebrio molitor*). Compared with baijiu lees (which are typically high in crude fiber and may predispose to gut inflammation) and brewer’s spent grain (which contain relatively high lignin and are less palatable), yellow wine lees differ in origin and composition. Yellow wine lees are not produced by distillation and are not mixed with fibrous additives; they tend to have low crude fiber and retain residual starch, protein and small fermentation metabolites (e.g., organic acids and *γ*-aminobutyric acid), attributes that render them nutrient-rich and relatively amenable to biological conversion (10–12). For example, Yao et al. (13) used fermented yellow wine lees (FYWL) as a dietary supplement for Holstein cows and found that milk from the FYWL group had significantly higher relative contents of mono-unsaturated fatty acids (MUFA, 36.90%), poly-unsaturated fatty acids (PUFA, 5.63%) and essential amino acids (EAA, 2.01%) compared with controls; Yao et al. (14) similarly reported that yellow wine lees can serve as a protein source for lactating cows and may improve herd profitability. Collectively, these characteristics suggest that yellow wine lees could be a suitable supplemental feedstock for saprophagous insects. To our knowledge, however, no study has systematically evaluated the use of yellow wine lees in the rearing of *H. illucens* larvae, particularly with respect to larval growth performance, nutrient accumulation, and gut microbiota composition. In the present study, we fed *H. illucens* larvae diets with graded inclusion levels of yellow wine lees and systematically examined growth performance (mean dry weight and substrate-reduction rate), larval nutritional composition (crude protein and ether extract) and gut microbial diversity and community structure. Our objective was to determine the dose–response relationship between dietary yellow wine lees inclusion and larval performance, and to explore the associations between gut microbiota and diet-induced changes in larval growth and nutrient composition, thereby providing a theoretical basis for the application of yellow wine lees in *H. illucens* rearing.

## 2 Materials and methods

### 2.1 Experimental materials

Black soldier fly eggs (Southeast Asian strain) were supplied by Guangzhou Wuliang Biotechnology Co., Ltd. Fresh eggs (<24 h old) from the same batch were used in all experiments and stored at 10 °C prior to incubation. Dietary ingredients included cornmeal (Shanxi Dafeng Seed Industry Co., Ltd.), wheat bran (Xinjishi Fuzhiyuan Flour Industry Co., Ltd.) and yellow wine lees (Shanxi Xinghua Jinshanbao Distillery Co., Ltd.).

### 2.2 Experimental instruments

ZDDN-2 Kjeldahl nitrogen analyzer (Zhejiang Top Cloud Agriculture Technology Co., Ltd.); TP-AD04 digestion/muffle furnace (Zhejiang Top Cloud Agriculture Technology Co., Ltd.); Q/WTCI03-2020 drying oven (Shanghai Precision Instrument Co., Ltd.); JC-ST-06(F) Soxhlet extractor (Qingdao Juchuang Environmental Protection Group Co., Ltd.); JY-057 vacuum freeze dryer (Wenzhou Jinbang Light Industry Machinery Co., Ltd.); and JXFM110 pulverizer (Hangzhou Keming Optoelectronics Technology Co., Ltd.). DNA extraction was performed using the FastDNA Spin Kit (Abbot Medical Device Trading Shanghai Co., Ltd.).

### 2.3 Experimental feed

Different proportions of yellow wine lees were incorporated into a cornmeal-wheat bran base (cornmeal:wheat bran = 1:1, w/w) to produce six experimental diets: T0 (control)-100% base; T1-90% base+10% yellow wine lees; T2-80% base+20% yellow wine lees; T3-70% base+30% yellow wine lees; T4-60% base + 40% yellow wine lees; and T5-50% base+50% yellow wine lees. Mixed diets were stored at −4 °C and used on the day of preparation. The composition and proximate nutritional levels of the experimental diets are reported in Table 1. Determination methods for crude protein (CP), ether extract (EE), ethanol content, and pH are described in Sections 2.5.4–2.5.6.

**Table 1:** Composition and nutritional levels of experimental diets (as-fed basis).

Items	Treatments					
	T0	T1	T2	T3	T4	T5
Ingredients						
Corn meal	50	45	40	35	30	25
Wheat bran	50	45	40	35	30	25
yellow wine lees	0	10	20	30	40	50
Total	100	100	100	100	100	100
Nutrient levels						
Crude protein	11.97	12.61	13.25	13.90	14.54	15.18
Ether extract	4.10	3.94	3.85	3.72	3.60	3.49
Ethanol content	0	0.40	0.81	1.20	1.61	2.04
pH value	6.33	5.95	5.51	5.07	4.64	4.20

Nutrient levels were measured values.

### 2.4 Test method

#### 2.4.1 Insect egg hatching

Five hundred grams of wheat bran were weighed and evenly spread in a plastic rearing box (30 cm × 20 cm × 10 cm). Distilled water was added to adjust the hatching substrate moisture to 75% (w/w). Eggs were evenly distributed over the substrate surface, and the box was incubated in a climatic incubator at 28 °C and 75% relative humidity in darkness. Hatching was checked daily. Newly hatched larvae from the same hatching period were collected and reared under the same environmental conditions for an additional 4 days before being used in the experiments.

#### 2.4.2 Experimental design

A total of 3,600 four-day-old black soldier fly larvae with similar initial body weight, standardized based on the mean weight of 100 randomly selected larvae, were selected and randomly allocated to six dietary treatments. Each treatment comprised three biological replicates of 200 larvae (*n* = 3 replicates per treatment; 200 larvae per replicate), and all biological replicates were analyzed independently. Each replicate received 250 g of feed, and the experimental diet moisture was adjusted to 60% (w/w). Larvae were maintained under the rearing conditions described above and the trial was terminated when prepupae first appeared; the experiment lasted 16 days. The study was performed in the training room of the Department of Biological Science and Technology, Jinzhong University, in March 2025.

### 2.5 Indicator detection

#### 2.5.1 Growth indicators and feed reduction rate measurement

Following the method described in (15), larvae and frass were separated at the end of the trial. Larvae were collected and lyophilized until a constant dry weight was obtained, after which the total larval dry weight and the total number of larvae per replicate were recorded. The mean larval dry weight was calculated as the total larval dry weight divided by the total number of larvae. Feed and the remaining residue (frass plus unconsumed substrate) were oven-dried at 100 °C until a constant weight was reached and weighed. Feed reduction rate (FR) on a dry-matter basis was then calculated as:


$$\text{Feed reduction}\;(\%)=\frac{\begin{array}{l} \text{Total}\;\mathrm{dry}\;\text{weight of feed}\;(g)–\\ \text{Residual solid}\;\mathrm{dry}\;\text{weight}\;(g) \end{array}}{\text{Total}\;\mathrm{dry}\;\text{matter of feed}\;(g)\times 100}\;$$ (1)

#### 2.5.2 Feed ingredients and analysis of insect body nutrients

The freeze-dried larvae were milled to a fine powder, passed through a 60-mesh (≈250 μm) stainless-steel sieve, and stored at −20 °C until analysis. Crude protein (CP) and ether extract (EE) in the larval biomass were determined according to the methods described in Sections 2.5.4 and 2.5.5, respectively.

#### 2.5.3 Detection of gut microbiota diversity in black soldier fly larvae

Sample pretreatment. Larvae were placed in an ice bath for 3–5 min. They were wiped with 70% (v/v) ethanol for 30 s, immersed in 0.25% (v/v) sodium hypochlorite for 1 min, and rinsed three times with sterile water. Gut dissection. Under aseptic conditions, each larval abdomen was incised and the gut was removed. Gut tissue was rinsed twice with sterile 0.9% (w/v) saline and transferred to sterile 1.5 mL microcentrifuge tubes. Ten gut samples were collected per group and stored at −80 °C. High-throughput sequencing. DNA was extracted using the FastDNA Spin Kit according to the manufacturer’s instructions. The V3-V4 regions of the bacterial 16S rRNA gene were amplified using the universal primer pairs 338F (5′-ACTCCTACGGGAGGCAGCAG-3′) and 806R (5′-GGACTACHVGGGTWTCTAAT-3′). PCR products were purified and quantified, and paired-end sequencing (2 × 300 bp) was performed on an Illumina MiSeq platform (2 × 300 bp; Illumina, San Diego, CA, United States). The use of 16S rRNA sequencing for culture-independent microbial community characterization has been described in both insect microbiome and broader microbiological studies (35, 36).

#### 2.5.4 Determination of crude protein

Crude protein (CP) content was determined by the Kjeldahl method according to GB/T 6432—2018, Determination of crude protein in feed (16). Samples were digested with concentrated sulfuric acid to convert organic nitrogen into ammonium salts in the presence of a catalyst. After alkalization and distillation, nitrogen content was quantified by standard acid titration, and CP content was calculated using a nitrogen-to-protein conversion factor of 6.25. The calculation formula was as follows:


$$\mathrm{CP}(\%)=\frac{(V_{1}–V_{0})\times c\times 14.007\times 6.25}{m\times 1000}\times 100$$ (2)

where *V*_1_ is the volume of standard acid consumed by the sample (mL); *V*_0_ is the volume of standard acid consumed by the blank (mL); *c* is the concentration of the standard acid (mol/L); and *m* is the sample mass (g).

#### 2.5.5 Determination of ether extract

Ether extract (EE) content was determined by the Soxhlet extraction method according to GB/T 6433—2006, Determination of ether extract in feed (17). Samples were continuously extracted with anhydrous ether, after which the solvent was removed and the extract was dried to constant weight. EE content was calculated as follows:


$$\mathrm{EE}(\%)=\frac{m_{1}–m_{0}}{m}\times 100$$ (3)

where *m*_1_ is the mass of the extraction flask after extraction (g); *m*_0_ is the mass of the empty extraction flask before extraction (g); and *m* is the sample mass (g).

#### 2.5.6 Determination of ethanol content and pH

Ethanol content was determined according to GB 5009.225—2023, Determination of ethanol in foods (18), using gas chromatography (GC). pH was determined according to GB 5009.237—2016, Determination of pH in foods (19). Samples were homogenized with distilled water at a ratio of 1:10 (w/v), and the pH of the homogenate was measured using a pH meter.

### 2.6 Data statistics and analysis

Growth performance, feed reduction rate, crude protein, ether extract, ethanol content, and pH were analyzed by one-way ANOVA using SPSS 19.0, followed by Fisher’s least significant difference (LSD) test for multiple comparisons. Linear and quadratic regression analyses were applied to assess the relationships between dietary yellow wine lees inclusion levels and larval growth performance and nutritional composition. Alpha-diversity indices, including Chao1, ACE, Shannon, and Simpson indices, were calculated to evaluate gut microbial richness and diversity. Microbial *β*-diversity was evaluated based on Bray–Curtis dissimilarity and UniFrac distance matrices. Pairwise Spearman rank correlations (r) between dominant microbial taxa and larval physiological indicators were computed in R. All results are presented as mean ± standard deviation, and statistical significance was accepted at *p* < 0.05. Figures and spatial mapping were generated using Origin 2024.

## 3 Results

### 3.1 Effect of yellow wine lees on the average dry weight and feed reduction rate of black soldier fly larvae

As presented in Table 2, the proportion of yellow wine lees had a significant effect on the growth performance of black soldier fly larvae (*p* < 0.05). Larvae in group T3 (30% inclusion) achieved the highest average dry weight (83.69 mg), which was significantly greater than that of the control (T0) and all other treatment groups (*p* < 0.05). In contrast, the average dry weights in groups T4 (40%) and T5 (50%) declined to 58.88 mg and 39.79 mg, respectively, both significantly lower than the control (*p* < 0.05). Feed-reduction rates did not differ significantly among groups T1 to T3 (65.05–68.49%); however, they decreased markedly in T4 (56.86%) and T5 (44.30%), with both values significantly lower than those of the remaining treatments (*p* < 0.05). Regression analysis indicated that larval dry weight exhibited a significant quadratic response to the proportion of yellow wine lees, whereas the feed-reduction rate declined in both linear and quadratic patterns (*p* < 0.05).

**Table 2:** Effects of yellow wine lees on average dry weight and feed reduction rate of black soldier fly larvae.

Items	Treatments					*p*-value			
	T0	T1	T2	T3	T4	T5	ANOVA	Linear	Quadratic
Average dry weight of larvae/mg	70.27 ± 2.24b	71.30 ± 2.55b	75.79 ± 1.03b	83.69 ± 2.07a	58.88 ± 3.39c	39.79 ± 1.80d	<0.001	0.006	<0.001
Feed reduction rate/%	65.05 ± 0.67a	65.82 ± 1.67a	67.37 ± 0.87a	68.49 ± 0.90a	56.86 ± 0.44b	44.30 ± 0.95c	<0.001	<0.001	<0.001

In the same row, data marked with different lowercase letters indicate significant differences (*p* < 0.05), while data marked with the same lowercase letters or without letters indicate no significant differences (*p* > 0.05). The same as below.

### 3.2 Effects of yellow wine lees on the crude protein and ether extract content of black soldier fly larvae

As shown in Table 3, the inclusion level of yellow wine lees significantly influenced the crude protein (CP) and ether extract contents of the larvae (*p* < 0.05). CP was highest in groups T2 and T3, reaching 48.37 and 47.96%, respectively, both significantly higher than the control group (39.16%) and groups T1, T4, and T5 (*p* < 0.05). Regression analysis further indicated a significant quadratic relationship between CP content and the proportion of yellow wine lees added (*p* < 0.05). Ether extract content exhibited a significant increasing trend with higher proportions of yellow wine lees, supported by both linear and quadratic regression analyses (*p* < 0.05). Group T5 showed the highest ether extract content (27.49%), which was significantly greater than that observed in all other treatment groups (*p* < 0.05).

**Table 3:** Effects of yellow wine lees on CP and EE contents on black soldier fly larvae.

Items	Treatments					*p*-value			
	T0	T1	T2	T3	T4	T5	ANOVA	Linear	Quadratic
CP	39.16 ± 0.56c	40.51 ± 1.52bc	48.37 ± 0.74a	47.96 ± 0.38a	42.93 ± 2.56b	43.76 ± 0.67b	<0.001	0.104	0.001
EE	12.03 ± 0.64d	12.51 ± 0.54d	16.13 ± 1.00c	19.04 ± 1.60bc	22.00 ± 1.12b	27.49 ± 2.91a	<0.001	<0.001	<0.001

### 3.3 Effects of yellow wine lees on the gut microbiota of black soldier fly larvae

#### 3.3.1 Analysis of gut microbiota diversity in black soldier fly larvae

As presented in Table 4, the proportion of yellow wine lees significantly affected the gut microbial diversity of black soldier fly larvae (*p* < 0.05). The Chao richness index did not differ significantly between the control group and groups T1-T3 (*p* > 0.05). However, when the inclusion level exceeded 40%, the Chao index declined significantly, with group T5 showing the lowest richness (*p* < 0.05). The Shannon index, reflecting both richness and evenness, increased with yellow wine lees up to 30%, reaching its highest value in group T3; nevertheless, this increase was not statistically different from the control, T1, or T2 (*p* > 0.05). At inclusion levels above 40%, the Shannon index declined markedly, and group T5 exhibited the lowest diversity (*p* < 0.05).

**Table 4:** Analysis of gut microbial diversity indices in black soldier fly larvae.

Items	Treatments					*p*-value			
	T0	T1	T2	T3	T4	T5	ANOVA	Linear	Quadratic
Chao richness index	109.71 ± 4.31a	111.97 ± 4.17a	108.93 ± 0.74a	104.52 ± 1.89a	86.22 ± 5.32b	70.29 ± 4.35c	<0.001	<0.001	0.001
Shannon index	2.55 ± 0.16a	2.58 ± 0.11a	2.67 ± 0.23a	2.73 ± 0.12a	2.04 ± 0.20b	1.62 ± 0.17c	<0.001	0.001	0.002

**Figure 1:**
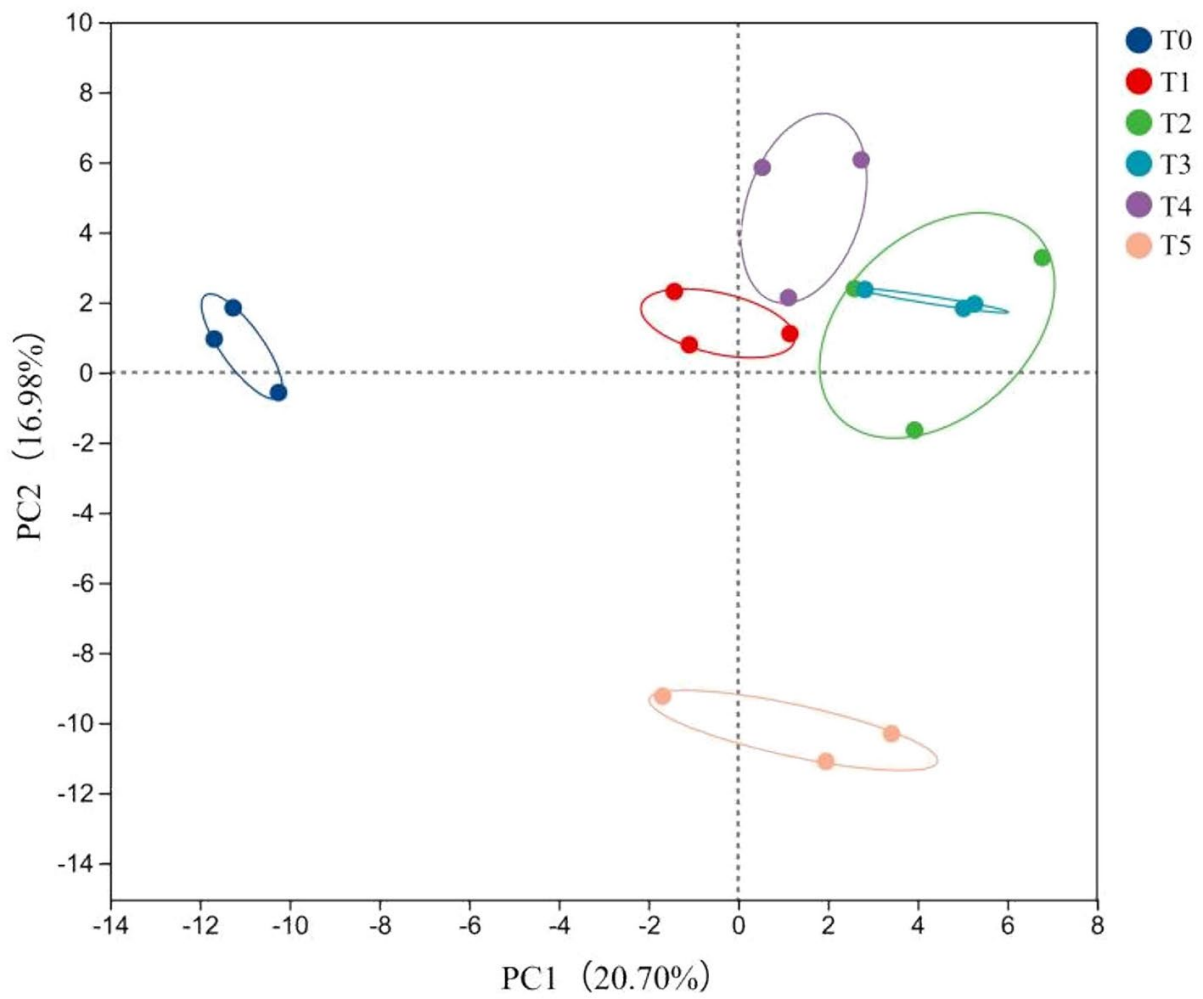
Principal coordinates analysis of gut flora in black soldier fly larvae.

As shown in Figure 1, principal coordinates analysis (PCoA) demonstrated distinct separation in the gut microbial community structures of larvae fed different proportions of yellow wine lees. Groups T2 and T3 formed a tight cluster in the primary coordinate space and were clearly separated from the control (T0) as well as from groups T1, T4, and T5, indicating that diets containing 20–30% yellow wine lees markedly shifted gut-microbiota composition relative to both the control and other treatments. The control group overlapped variably with T1, T4, and T5 across comparisons, suggesting that the microbial communities in the low-addition group (10%) and in the high-addition groups (40 and 50%) also diverged significantly from those of the other treatments.

#### 3.3.2 Relative abundance at the phylum level of gut microbiota

As shown in Table 5, the dominant bacterial phyla in the gut tract of black soldier fly larvae were *Firmicutes* and *Proteobacteria*, together accounting for more than 80% of the total community. The relative abundance of *Firmicutes* was significantly higher in groups T2 and T3 than in all other treatments (*p* < 0.05), whereas no significant differences were observed among the control (T0), T1, T4, and T5 (*p* > 0.05). *Firmicutes* exhibited a significant quadratic response to the proportion of yellow wine lees added (*p* < 0.05). *Proteobacteria* abundance in T2 and T3 was significantly lower than in the control and remaining groups (*p* < 0.05), following a pattern of initial decline and subsequent increase (*p* < 0.05). *Bacteroidetes* abundance was significantly higher in T2, T3, and T5 than in the control and other treatments (*p* < 0.05), although the quadratic trend was not significant (*p* > 0.05). *Actinobacteria* abundance was significantly elevated in group T1 compared with all other groups (*p* < 0.05) and decreased linearly with increasing proportions of yellow wine lees (*p* < 0.05). *Clostridia* showed significant linear and quadratic increases across treatments (*p* < 0.05), reaching the highest abundance in T5, while being detected only in trace amounts in the control and in groups T1–T3. Cyanobacteria were detected in the control group (0.46%) but were absent or present only at trace levels in all treatment groups.

**Table 5:** Effects of yellow wine lees on relative abundance on gut flora at phylum level in black soldier fly larvae.

Item	Treatment					*p*-value			
	T0	T1	T2	T3	T4	T5	ANOVA	Linear	Quadratic
*Firmicutes*	38.64 ± 1.44b	40.03 ± 2.11b	56.93 ± 2.39a	56.85 ± 2.87a	41.44 ± 1.98b	44.95 ± 2.91b	<0.001	0.376	0.005
*Proteobacteria*	54.17 ± 2.19a	49.92 ± 3.80a	36.43 ± 1.33b	34.68 ± 1.34b	52.49 ± 2.03a	46.91 ± 5.76a	<0.001	0.463	0.009
*Bacteroidota*	2.66 ± 1.70ab	1.98 ± 1.58b	4.27 ± 0.31a	6.18 ± 0.19a	3.04 ± 0.73ab	5.42 ± 0.36a	0.002	0.022	0.059
*Actinomycetota*	3.95 ± 0.18b	8.01 ± 0.27a	2.38 ± 0.12c	2.13 ± 0.05c	2.47 ± 0.48c	1.31 ± 0.18d	<0.001	0.003	0.014
*Fusobacteriota*	0.01 ± 0.00c	0.00 ± 0.00c	0.00 ± 0.00c	0.00 ± 0.00c	0.52 ± 0.11b	1.35 ± 0.19a	<0.001	<0.001	<0.001
*Cyanobacteriota*	0.46 ± 0.03a	0.00 ± 0.00b	0.00 ± 0.00b	0.00 ± 0.00b	0.00 ± 0.00b	0.00 ± 0.00b	<0.001	0.003	<0.001

#### 3.3.3 Relative abundance at the genus level of gut microbiota

As shown in Table 6, the gut microbiota at the genus level differed markedly among larvae fed different proportions of yellow wine lees (*p* < 0.05). In group T1, only *Lactobacillus* increased significantly relative to the control (p < 0.05), while other genera showed no significant changes (*p* > 0.05). In T2, *Klebsiella* abundance was significantly lower than in the control and in groups T1, T4, and T5 (*p* < 0.05). *Enterococcus* also decreased significantly, whereas *Terrisporobacter* increased to 12.97%, significantly exceeding the control and groups T1, T4, and T5 (*p* < 0.05). *Enterobacter* reached the highest abundance among all groups, while *Prodensiella* showed a significant reduction. *Lactobacillus* was also significantly higher than in the control and T1 (*p* < 0.05). In T3, *Terrisporobacter* exhibited the highest abundance across all treatments, while *Enterococcus* and *Prodensiella* were at their lowest levels. *Klebsiella* remained significantly lower than in the control and T4 (*p* < 0.05). *Sphingobacterium* was significantly enriched compared with the control and groups T1 and T4, and *Lactobacillus* remained significantly elevated relative to the control and T1 (*p* < 0.05). In T4, *Klebsiella* rebounded to a level not significantly different from the control (*p* > 0.05). *Enterococcus* did not differ from T2 (*p* > 0.05) but remained significantly lower than the control (*p* < 0.05). In T5, *Clostridium* reached the highest abundance among all groups and was significantly higher than in the control and groups T1–T3 (*p* < 0.05). *Lactobacillus* also remained significantly elevated compared with the control and T1-T3 (*p* < 0.05). Meanwhile, *Enterococcus* and *Prodensiella* returned to levels comparable to the control (*p* > 0.05). *Terrisporobacter* declined significantly, becoming the lowest among all groups (*p* < 0.05). *Fusobacterium* was significantly higher than in T4 (*p* < 0.05).

**Table 6:** Effects of yellow wine lees on relative abundance on gut flora at genus level in black soldier fly larvae.

Item	Treatment					*p*-value			
	T0	T1	T2	T3	T4	T5	ANOVA	Linear	Quadratic
*Klebsiella*	25.34 ± 3.28a	22.33 ± 2.82a	9.98 ± 3.15c	15.90 ± 3.27b	25.72 ± 3.09a	17.50 ± 3.98ab	<0.001	0.471	0.135
*Enterococcus*	16.24 ± 2.99a	15.85 ± 1.91a	8.87 ± 1.08b	3.06 ± 1.58c	7.93 ± 4.20b	13.86 ± 2.19a	<0.001	0.110	0.001
*Enterobacter*	14.73 ± 1.41b	14.16 ± 1.27b	19.77 ± 1.31a	14.41 ± 2.55b	15.52 ± 0.66ab	12.39 ± 1.34b	0.002	0.328	0.044
*Weissella*	10.52 ± 2.55a	11.16 ± 0.79a	15.87 ± 2.87a	12.34 ± 2.18a	13.67 ± 1.78a	16.00 ± 3.37a	0.066	0.024	0.080
*Terrisporobacter*	6.09 ± 0.80c	6.17 ± 0.78c	12.97 ± 2.21b	17.46 ± 0.45a	5.91 ± 0.60c	2.11 ± 0.56d	<0.001	0.550	<0.001
*Lachnoclostridium*	1.66 ± 0.13c	1.63 ± 0.12c	3.97 ± 1.38c	6.32 ± 1.14bc	10.68 ± 0.48b	16.40 ± 3.46a	<0.001	<0.001	<0.001
*Providencia*	13.99 ± 3.89a	13.19 ± 2.45a	6.41 ± 0.81b	3.66 ± 0.71c	10.71 ± 1.23ab	16.08 ± 0.85a	<0.001	0.992	<0.001
*Sphingobacterium*	2.04 ± 0.58b	1.78 ± 0.37b	4.03 ± 1.74a	5.93 ± 0.48a	2.91 ± 0.99b	5.35 ± 0.77a	0.001	0.008	0.020
*Lactiplantibacillus*	0.05 ± 0.02c	2.77 ± 1.41b	4.18 ± 0.87ab	4.78 ± 0.49a	5.67 ± 0.46a	5.73 ± 0.38a	<0.001	<0.001	<0.001
*Fusobacterium*	0.01 ± 0.00b	0.00 ± 0.00b	0.00 ± 0.00b	0.00 ± 0.00b	0.43 ± 0.16ab	1.34 ± 0.38a	<0.001	<0.001	<0.001

#### 3.3.4 Correlation between gut microbiota and growth and nutritional indicators

**Figure 2:**
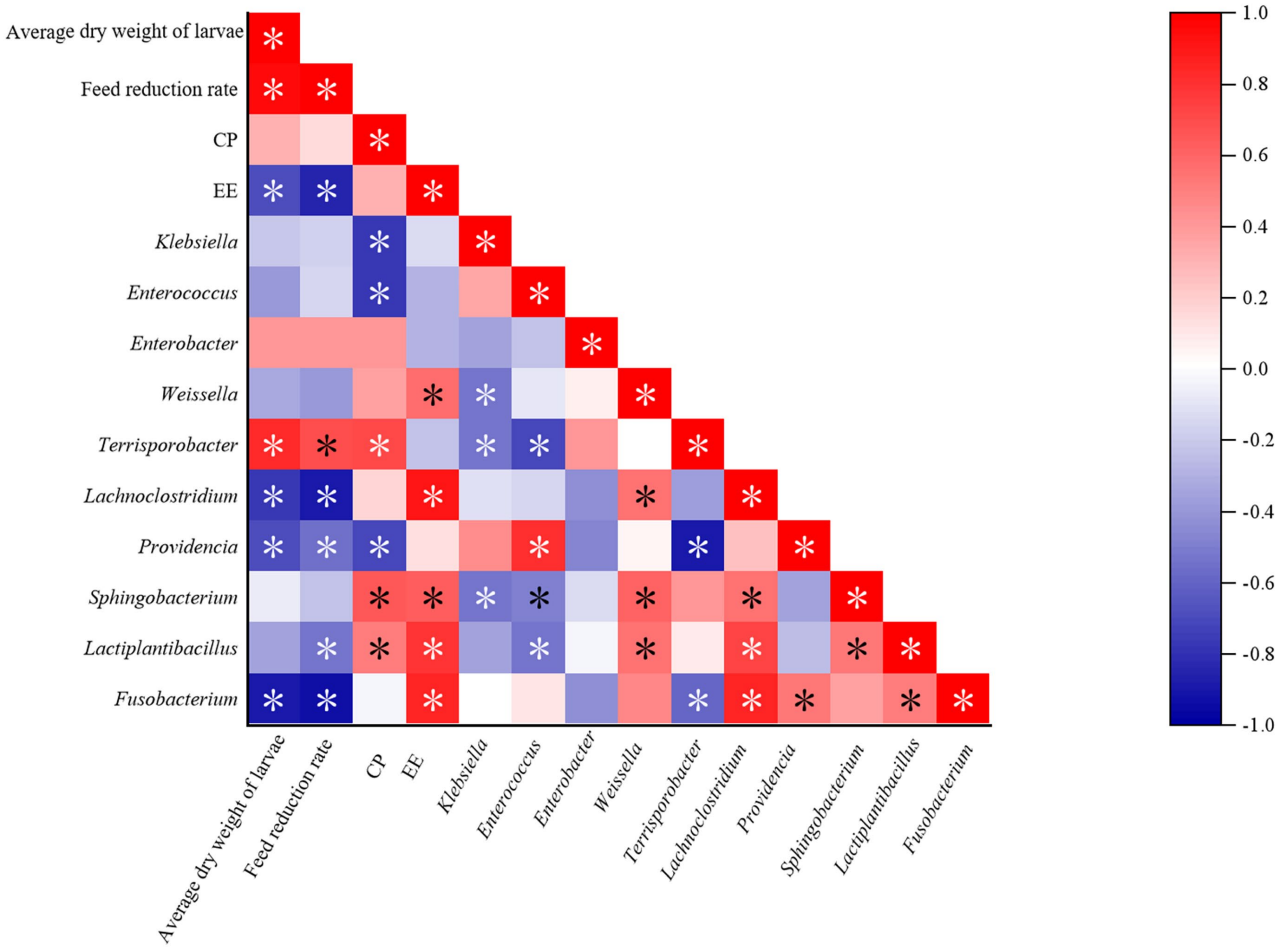
Correlation analysis of average dry weight, feed reduction rate, CP, EE content of black soldier fly larvae with relative abundance of main gut genera.

As shown in Figure 2, correlation analysis revealed significant associations between gut-microbiota genera and larval performance indicators. The relative abundance of *Terrisporobacter* was positively correlated with both average larval dry weight and feed-reduction rate (*p* < 0.05). In contrast, *Clostridium*, *Prodensiella*, and *Fusobacterium* were all negatively correlated with average dry weight (*p* < 0.05), and *Clostridium*, *Prodensiella*, and *Lactobacillus* also showed significant negative correlations with feed-reduction rate (*p* < 0.05). *Terrisporobacter*, *Sphingobacterium*, and *Lactobacillus* were positively correlated with larval crude protein content, whereas *Klebsiella*, *Enterococcus*, and *Prodensiella* exhibited significant negative correlations (*p* < 0.05). Additionally, *Weissella*, *Clostridium pilosa*, *Sphingobacterium*, *Lactobacillus*, and *Fusobacterium* displayed significant positive correlations with ether extract content (*p* < 0.05). It should be noted that the correlations between microbial genera and larval growth and nutritional indicators were derived from correlation analysis across dietary treatments and may have been simultaneously influenced by coordinated changes in dietary crude protein content, ethanol concentration, and pH. Therefore, these results only reflect potential associations between gut microbiota and host phenotypes, rather than direct causal effects of yellow wine lees.

## 4 Discussion

### 4.1 Effect of the proportion of yellow wine lees added on the growth of black soldier Fly larvae

Parra et al. (20) conducted a feeding trial in which black soldier fly larvae (BSFL) were reared on bovine manure, ovine manure, and mixtures of agave bagasse and vinasse, a by-product of mezcal distillation. Their results showed that larvae fed sheep manure achieved the highest growth performance, whereas those reared on the agave bagasse-vinasse mixture exhibited the lowest growth rates. Similarly, Chia et al. (21) reported that the inclusion of brewer’s grains significantly increased larval weight. Li et al. (22) further demonstrated that incorporating 45% wet wine lees into cow or pig manure increased the waste-reduction rate by 20% and improved the bioconversion efficiency of BSFL by 10%. Compared with other distillers’ residues, yellow wine lees are characterized by lower crude fiber and higher concentrations of readily available nutrients. Such properties suggest that yellow wine lees may influence the growth performance and nutritional composition of saprophagous insects. In this study, both average larval dry weight and feed-reduction rate were maximized at a 30% inclusion level (T3), significantly exceeding the control and all other treatments. This bell-shaped dose–response, reflected by a significant quadratic regression, indicates that moderate supplementation with yellow wine lees (20–30%) can effectively enhance larval growth. Yao et al. (23) also reported beneficial physiological effects of yellow wine lees in livestock: feeding Holstein cows a total mixed ration containing 11% yellow wine lees reduced oxidative stress in heat-stressed animals and improved the oxidative stability of their milk, compared with a ration containing 18% soybean meal. Together with our findings, these results suggest that the growth-promoting effects observed in diets containing appropriate levels of yellow wine lees may derive from its residual starch, protein, and micronutrients, as well as small-molecule fermentation metabolites that provide readily utilizable energy and nutrients for larval development.

However, when the inclusion level exceeded 30% (groups T4 and T5), both larval dry weight and feed-reduction rate declined significantly. This inhibitory effect was closely associated with changes in the physicochemical properties introduced by diets containing high proportions of yellow wine lees. First, ethanol content increased markedly, reaching 2.04% in T5. Previous studies have shown that chronic ethanol exposure elevates colonic oxidative-stress markers and pro-inflammatory cytokines, alters immune-cell populations-including reductions in regulatory T cells-and increases advanced glycation end-products (AGEs) and their receptor RAGE, ultimately contributing to gut lesions (24). Moreover, accumulating evidence indicates that alcohol intake disrupts gut microbial homeostasis, and ethanol-induced dysbiosis is considered an early driver of alcohol-related disorders such as alcohol use disorder and alcoholic liver disease (25). Second, feed acidification intensified substantially (pH 4.20 in T5). Low pH (≤4.0) has been reported to reduce BSFL larval weight by approximately 23% compared with neutral controls (26). Strongly acidic conditions may impair gut physiology, reduce the activity of key digestive enzymes (e.g., proteases, lipases), and consequently hinder nutrient assimilation. Additionally, a high proportion of yellow wine lees may alter dietary texture-such as increasing viscosity or reducing palatability-which in turn disrupts normal feeding behavior and digestion. Collectively, these findings indicate that inclusion levels above 40% constitute a critical threshold beyond which larval growth is inhibited, and therefore should be avoided in practical feeding applications.

### 4.2 Effect of the addition ratio of yellow wine lees on the nutritional components of insects

Aam et al. (27) found that adding varying proportions of lemuru fish oil to laying-hen manure used as substrate for black soldier fly larvae significantly affected both larval weight and ether extract content. Wang et al. (28) reported that inclusion of oilseed meals raised larval crude protein from 9.24 to 14.35% and ether extract from 18.6 to 21.95%, underlining the strong relationship between diet composition and larval nutrient profiles. In the present study, supplementation with yellow wine lees markedly altered larval crude protein and fat deposition: the medium-proportion groups (T2, T3) attained the highest crude protein levels (48.37–47.96%), which coincided with maximal growth. This pattern suggests that moderate inclusion of yellow wine lees promotes efficient conversion of dietary nutrients into high-quality larval protein, enhancing their value as a protein feed or processing raw material. By contrast, ether extract content increased in a near-linear manner with greater lees inclusion, reaching 27.49% in T5. Such an increase may reflect the presence of lipid precursors (for example, soluble sugars or fermentation-derived organic acids) in the lees that are readily channeled into *de novo* lipid synthesis, and it may also be related to enrichment of taxa such as *Lactobacillus* and *Fusobacterium* in high-inclusion groups, which have been implicated in host lipid-metabolic pathways. Zhong (29) reported that polyphenols from distillers’grains can suppress opportunistic pathogens, increase *Lactobacillus* abundance in the gut and alter fatty-acid profiles, consistent with our observations. However, the highest fat content was accompanied by substantially reduced growth performance (lowest dry weight in T5), suggesting that, at high inclusion levels, larvae may divert energy toward fat storage or invoke a stress-related metabolic strategy rather than somatic growth. These results indicate a trade-off between tailoring body-component profiles and maintaining growth efficiency that should be considered when optimizing diets for specific production goals.

### 4.3 Effect of the proportion of yellow wine lees added on gut microbiota

Studies have shown that replacement of part of the basal diet with fermented yellow wine lees can significantly improve growth performance, meat quality and immune status in finishing pigs, and can alter the gut microbiota by increasing the abundance of beneficial bacteria such as *Lactobacillus* (30). In the present study, different dietary inclusion levels of yellow wine lees induced significant changes in gut microbiota structure and diversity, and these changes were significantly correlated with differences in larval growth and body composition, suggesting that the gut microbiota may play an important mediating role in these responses. The Chao richness index in the low and medium-proportion groups (T1–T3) did not differ from that of the control, indicating that species richness remained stable; however, the high-proportion groups (T4, T5) exhibited a significant decrease in richness, indicating species loss when the inclusion level reached ≥40%. The Shannon diversity index peaked in T3 and was lowest in T5, confirming that high inclusion levels disrupted gut-microbiota balance. Loss of microbial species and structural imbalance may reduce gut stability and health, thereby adversely affecting the growth and metabolism of *Hermetia illucens* larvae.

#### 4.3.1 Optimizing microbial community structure and functional adaptation with low-proportion addition of yellow wine lees

PCoA analysis revealed that the gut microbiota structure of the medium- and low-proportion addition groups (T2 and T3) was clearly separated from that of the control and the other groups. The phylum *Firmicutes*-particularly the genus *Terrisporobacter*-was enriched in T2 and T3 and showed strong positive correlations with mean larval dry weight, feed-reduction rate and crude-protein content. In contrast, communities enriched in the phylum *Proteobacteria* and genera such as *Klebsiella* and *Prodensiella* were negatively correlated with growth and protein accumulation. Hao et al. (15) similarly found that dietary addition of purple-potato-skin powder increased *Terrisporobacter* abundance in the BSFL gut; this increase correlated positively with crude-protein content and negatively with waste-reduction rate and ether extract content, which aligns with our observations. *Firmicutes* are widely recognized for their capacity to hydrolyze and ferment carbohydrates and other complex substrates, processes that can enhance anaerobic fermentation of fiber and starch residues and thereby support the growth advantages observed in T2 and T3. The concomitant reduction in *Proteobacteria* and several potential opportunistic genera (e.g., *Klebsiella*, *Prodensiella*) further indicates that a moderate inclusion of yellow-wine lees can optimize gut microecology by suppressing harmful taxa (30, 31). Overall, these shifts in community composition and function provide a plausible microbiota-mediated mechanism linking diet composition to larval nutrient conversion and growth performance (32, 33).

#### 4.3.2 High proportion of yellow wine lees addition leads to microbial imbalance and metabolic disorder

When the proportion of yellow wine lees in the diet was too high (T4, T5), gut-microbiota diversity and community balance declined markedly. This shift was accompanied by abnormal enrichment of *Fusobacterium* and *Clostridium*, which correlated positively with growth inhibition and excessive fat accumulation. Gao et al. (34) reported that supplementing the diet of Holstein cows with *Clostridium butyricum* increased short-chain fatty-acid levels and improved gut health. Such structural shifts indicate an imbalance in gut microecology in which these genera may favor fat-producing metabolic pathways. In addition, although *Lactobacillus* abundance increased in T4 and T5 and correlated positively with ether extract content, it was negatively associated with feed-reduction rate, suggesting that under high-inclusion conditions *Lactobacillus* may shift toward lipid-focused metabolism rather than supporting efficient nutrient utilization.

## 5 Conclusion

Fed diets in which yellow wine lees replaced 20–30% of corn flour and wheat bran, BSFL exhibited optimized gut microbial structure, enhanced larval growth, and increased larval crude protein content. In contrast, when the replacement level exceeded 40%, dietary acidification and ethanol accumulation were accompanied by gut microbial dysbiosis, severe growth inhibition, and excessive fat deposition in larvae. These changes in larval growth and metabolism were significantly correlated with alterations in gut microbial community structure, suggesting that the gut microbiota may play a mediating role in these responses. The present findings provide a scientific reference for determining appropriate inclusion levels of yellow wine lees and understanding microbiota-associated mechanisms in black soldier fly production.

## Data Availability

The original contributions presented in the study are included in the article/supplementary material, further inquiries can be directed to the corresponding author.
